# Remote Ischemic Preconditioning to Prevent Delayed Cerebral Ischemia Due to Vasospasm in the Treatment of Patients with Aneurysmal Subarachnoid Hemorrhage

**DOI:** 10.1007/s12028-025-02324-y

**Published:** 2025-07-15

**Authors:** Carolin Albrecht, Teresa Silvestri, Maria Wostrack, Christian Maegerlein, Insa Janssen, Jan Martin, Bernhard Meyer, Arthur Wagner, Jens Gempt

**Affiliations:** 1 Department of Neurosurgery, TUM School of Medicine and Health, Klinikum rechts der Isar, Technical University Munich, Munich, Germany; 2 Department of Anesthesiology and Intensive Care Medicine, TUM School of Medicine and Health, Klinikum rechts der Isar, Technical University Munich, Munich, Germany; 3 Department of Diagnostic and Interventional Neuroradiology, TUM School of Medicine and Health, Klinikum rechts der Isar, Technical University Munich, Munich, Germany; 4 Department of Neurosurgery, Hôpitaux Universitaires de Genève, Geneva, Switzerland; 5 Department of Neurosurgery, University Hospital Hamburg-Eppendorf, Hamburg, Germany

**Keywords:** Aneurysmal subarachnoid hemorrhage, Aneurysm rupture, RIPC, Remote ischemic preconditioning, Delayed cerebral ischemia, DCI

## Abstract

**Background:**

Remote ischemic preconditioning (RIPC) has shown potential in reducing vasospasm-induced secondary ischemia after aneurysmal subarachnoid hemorrhage (aSAH). Research suggests RIPC may help the brain adapt to periods of reduced blood flow, thereby reducing the risk of cerebral infarction secondary to delayed cerebral ischemia. This study aimed to analyze the possible impact of RIPC in patients with vasospasm following aSAH.

**Methods:**

We performed a prospective, randomized, controlled, and rater-masked trial at our high-volume neurovascular center. Patients treated for aSAH between November 2019 and September 2023 were randomly allocated to either the control or RIPC intervention group. The RIPC intervention involved three upper arm blood pressure cuff inflations (20 mm Hg above systolic pressure) for 5 min, followed by 5 min of reperfusion, administered for 10 consecutive days within the initial 14 days after aSAH. The primary end point was postinterventional computed tomography to identify new cerebral infarction areas.

**Results:**

Among 60 patients (29 in the intervention group, 31 in the control group) the entire cohort averaged 62.0 years, with no significant age difference between groups (*p* = 0.41). RIPC did not significantly affect the initial occurrence of symptomatic vasospasms or the incidence of cerebral infarctions (RIPC 24.1% vs. control 16.1%, *p* = 0.44). No significant difference was found between the two groups with respect to incidence of new neurological symptoms (*p* = > 0.99) or in-hospital mortality (*p* = 0.5).

**Conclusions:**

Remote ischemic preconditioning does not appear to influence the occurrence of vasospasms or the development of new infarcts on computed tomography. Larger studies are needed to further explore whether RIPC may have a role in specific high-risk subgroups or clinical settings.

## Introduction

Aneurysmal subarachnoid hemorrhage (aSAH) is a serious neurological disorder associated with high rates of morbidity and mortality [1]. aSAH typically results from the rupture of a cerebral aneurysm, leading to extravasation of blood into the subarachnoid space. This can trigger a cascade of pathological events, including cerebral vasospasm (CVS), delayed cerebral ischemia (DCI), and ultimately neuronal injury and death [2]. Current treatment approaches focus on occluding the ruptured aneurysm, typically by surgical clipping or endovascular coiling, and managing the various complications that can arise, such as hydrocephalus and DCI [2, 3].

An additional contributor to the cascade of injury is reperfusion damage, which occurs following vascular ischemia during DCI or secondary infarction, further exacerbating neuronal injury [4, 5]. This process is characterized by the generation of reactive oxygen species, activation of the complement system, adhesion of leukocytes to endothelial cells, aggregation of platelets and leukocytes, increased microvascular permeability, and impaired endothelium-dependent vasorelaxation [6, 7].

To prevent ischemia–reperfusion injury, remote ischemic preconditioning (RIPC), a therapeutic approach that involves short, controlled periods of ischemia followed by reperfusion in a tissue or organ has been widely studied [7–10]. This approach has been investigated particularly in the context of aortic and coronary artery bypass surgeries, in which it has demonstrated potential in reducing ischemia–reperfusion damage and improving clinical outcomes across various settings [8, 9, 11].

Given the critical impact of DCI and subsequent cerebral infarction on outcomes in patients with aSAH, our study investigated whether RIPC could reduce the occurrence of cerebral infarction as an objective surrogate marker of delayed ischemic injury. We hypothesized that RIPC provides neuroprotection by lowering the risk of cerebral infarction in this population.

## Methods

### Study Design and Cohort

This study was designed as a single-center, randomized, double-masked, parallel, two-group, controlled trial with a 1:1 allocation ratio.

Patients were included in the analysis based on the following criteria:

1. Admission with acute aSAH between November 2019 and October 2023.
2. Presence of a ruptured aneurysm, verified through computed tomography (CT) angiography or digital subtraction angiography, which was subsequently treated and occluded at our center.
3. Age > 18 years.

Patients were excluded if they were younger than 18 years, pregnant, unable to provide informed consent, had peripheral arterial disease, had diabetes mellitus requiring oral antidiabetic medication, or had symptom onset more than 48 h prior to admission.

### RIPC

The study included two groups: the RIPC (intervention) group and the control group. In the RIPC group, the intervention consisted of three inflations of a blood pressure cuff placed on the upper arm to 20 mm Hg above the patient’s systolic blood pressure. Each inflation lasted for 5 min, followed by a 5-min period of reperfusion during which the cuff was deflated. This procedure was repeated once daily for 10 consecutive days, starting after aneurysm treatment and within the first 14 days following the bleeding event (median 2 days after aneurysm securing, range 1–4 days). In the control group a blood pressure cuff was similarly placed on the upper arm, but no inflation or intervention was performed. This ensured that participants in both groups experienced the same conditions except for the active intervention.

### Primary Outcome

The primary end point was the occurrence of new cerebral infarctions identified on follow-up native CT scans 2 to 3 weeks after the bleeding event. These were conducted according to routine clinical protocol, with axial slices of 0.9 mm thickness prospectively evaluated by a senior neuroradiologist. Cerebral infarction was defined as a new area of hypodensity within the cerebral parenchyma in comparison to the routine CT scan on admission. Although not limited to a single reviewer, all evaluations were conducted within the same department and according to established clinical and diagnostic standards.

Clinical DCI, as defined by Vergouwen et al. [2], was not assessed as a primary end point because of confounding factors such as sedation, impaired consciousness, and fluctuating neurological status in this cohort.

### Secondary Outcomes

Secondary outcomes included the incidence rates and severity of new neurological deficits during in-hospital stay; transcranial Doppler (TCD) velocities measured daily between days 3 and 21 and modified Rankin Scale (mRS) scores recorded at hospital discharge and at the 6-month follow-up. Motor function was assessed using the Medical Research Council muscle strength grading system daily in the morning by an experienced neurologist. Flow velocities were recorded in the middle cerebral artery, anterior cerebral artery, and internal carotid artery. Vasospasm was defined as moderate if flow velocities were between 120 and 200 cm/s and as severe if they exceeded 200 cm/s.

### Ethical Approval

The study was conducted in compliance with the principles outlined in the Declaration of Helsinki. Approval for the study was granted by the Institutional Review Board of the Technical University of Munich (Reference number 336/16s). Written informed consent was obtained from every patient on study inclusion. In addition, the study had been registered in the German Clinical Studies Registry under registration number DRKS00011177 prior to study initiation.

### Sample Size

The sample size calculation for this explorative, single-center study was based on previous research, including a study by Sales et al. [12] and a randomized trial by Meng et al. [13]. Anticipating a reduction of more than 50% in the occurrence of new ischemic infarcts on postprocedural CT scans in the intervention group, the required sample size was estimated to be 24 patients per group. This calculation was based on a two-sided test with a significance level (α) of 0.05 and a statistical power of 80%. To account for potential dropouts and imbalances in patient allocation, additional participants were included, resulting in a planned enrollment of 30 patients per group.

In the absence of established effect sizes for RIPC in the aSAH population, a 50% risk reduction is used as a working assumption. Consequently, the study should be considered exploratory in nature, with its primary objective being the assessment of feasibility, safety, and preliminary signals of efficacy.

### Randomization and Masking

Participants were randomly assigned to either the RIPC group or the control group in a 1:1 ratio using a computer-generated randomization list. The randomization sequence was created, and group allocations were assigned by an independent researcher who was not involved in the treatment or outcome evaluation.

The assessors responsible for evaluating outcomes were masked to the group assignments.

### Statistical Analysis

Statistical analysis was performed using IBM SPSS Version 29.0.1.0. Categorical variables were analyzed with the Pearson *χ*^2^ test. Continuous variables were assessed for normality using the Shapiro–Wilk test. For normally distributed data, an independent samples *t*-test was applied to compare means between groups, whereas the Mann–Whitney *U*-test was used for nonnormally distributed data. Multivariable analysis was performed using logistic regression models for binary outcomes and linear regression models for continuous outcomes to adjust for potential confounders. Statistical significance was set at a *p* value < 0.05.

## Results

### Patient Demographics and Clinical Presentation

A total of 60 patients were included in the study, divided into a control group (*n* = 31) and an intervention group (*n* = 29). The baseline demographics and clinical characteristics were comparable across the groups. Table 1 summarizes the patient demographics indicating no significant differences between the groups.

**Table 1:** Patient demographics

Demographic	Group 1: control (*n* = 31)	Group 2: intervention (*n* = 29)	*p* value
Age, mean ± SD	62.8 ± 10.2	59.8 ± 12.3	0.41
Female (%)	74.2	62.1	0.46
GCS at admission, mean ± SD	7.7 ± 4.9	9.2 ± 5.6	0.278
Hunt & Hess grade (%)			
1	6.5	6.9	> 0.99
2	12.9	34.5	0.095
3	25.8	13.8	0.40
4	45.2	20.7	0.08
5	9.7	24.1	0.25
Fisher CT grade (%)			
1	0	0	
2	10.3	14.3	0.96
3	20.7	35.7	0.333
4	68.9	50	0.234
Localization (%)^a^			
BA/PCA/PComA	6.5	10.3	0.12
ACA/AComA	64.5	41.4	0.46
ACI	3.2	10.3	0.93
MCA	25.8	37.9	0.56
Intervention (%)			
Coiling	45.2	55.2	0.61
Clipping	54.8	44.8	0.61

ACA, anterior cerebral artery; ACI, internal carotid artery; AComA, arterior communicating artery; BA, basilar artery; CT, computed tomography; GCS, Glasgow Coma Scale; MCA, middle cerebral artery; PCA, posterior cerebral artery; PComA, posterior communicating artery; SD, standard deviation

^a^Distribution of aneurysm locations within each group

The location of the aneurysms showed a predominance in the anterior circulation (anterior cerebral artery, anterior communicating artery, and middle cerebral artery) in both groups, with no significant differences in the distribution between the groups (*p* = 0.31).

The most common symptom among both groups was a typical thunderclap headache reported by 60% of patients. This was followed by decreased vigilance in 43% of patients and nausea or vomiting in 32%. Other symptoms included meningism in 18% of patients, focal neurological deficits in 15%, and seizures in 12%. Among all, 17% of patients collapsed shortly after admission.

### Tolerability and Safety of RIPC

Remote ischemic preconditioning was initiated after completion of aneurysm treatment, with a median delay of 2 days (range 1–4 days) between aneurysm securing and the first session.

Overall, one patient (1.6%) experienced mild swelling of the arm, which resolved in under 5 min. Two other patients (3.2%) reported mild paresthesia in the affected upper limb, which also resolved. One of these patients also reported pain in the arm. Another patient experienced a headache after the RIPC session. No other side effects were observed.

### Primary Outcome: New Infarcts on CT

**Fig. 1:**
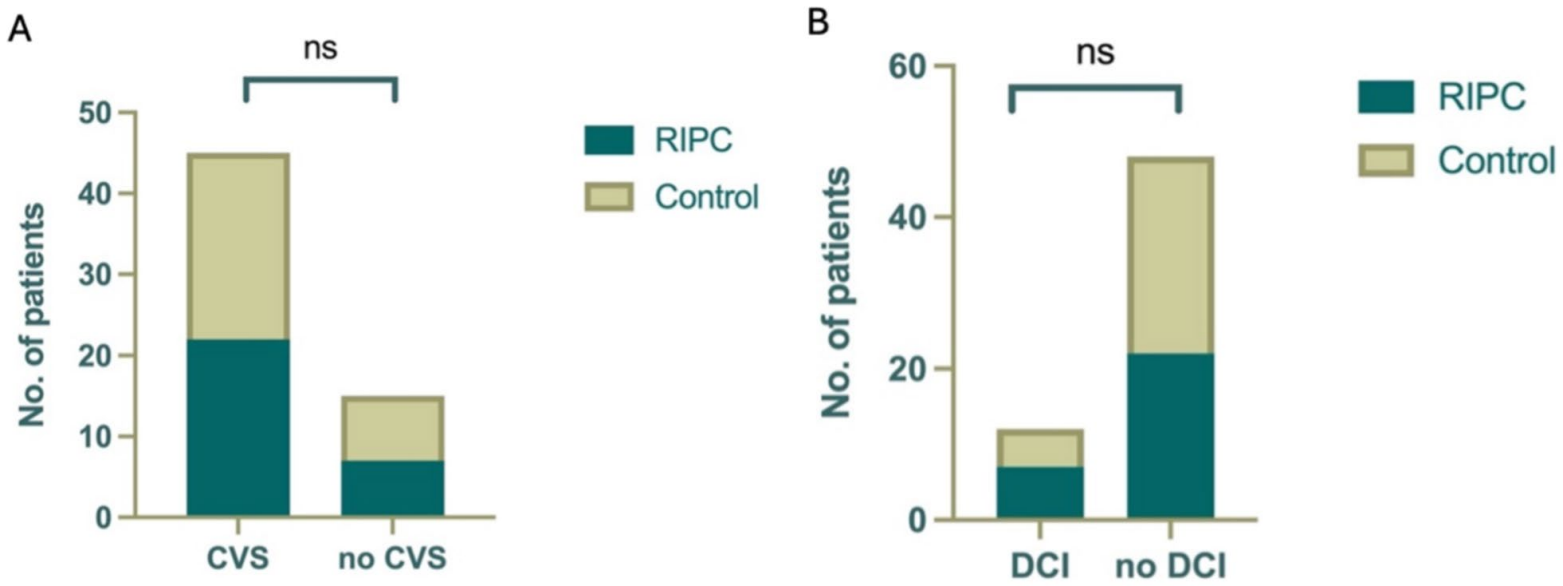
No difference in the occurrence of CVS measured by **a** TCD or **b** DCI was observed between the groups. CVS, cerebral vasospasm; DCI, delayed cerebral ischemia; ns, not significant; RIPC, remote ischemic preconditioning

Across all patients, 12 (20%) had new infarcts on postprocedural CT scans. RIPC did not demonstrate any neuroprotective effect, as both groups exhibited a similar occurrence rate of infarcts (RIPC 24.1% vs. control 16.1%; *p* = 0.44, *χ*^2^ test; Fig. 1b).

### Secondary Outcome: TCD Velocities

Altogether, TCD elevations were observed in 45 patients (75%). In the control group, eight patients (25.8%) had no significant TCD velocity elevations, whereas in the intervention group five patients (17.2%) did not experience TCD values above 120 cm/s (*p* = 0.57). Eight patients (25.8%) in the control group and 11 patients (37.9%) in the intervention group experienced moderate TCD elevations between 120 and 200 cm/s (*p* = 0.46). Vasospasm, defined as TCD above 200 cm/s, was observed in 15 patients in the control group (48.4%) and 11 patients in the interventional group (37.9%, *p* = 0.62). In the control group, 74.2% of patients experienced CVS, compared with 75.9% in the RIPC group. The difference in CVS occurrence between the groups was not statistically significant (*p* = 0.88, *χ*^2^ test; Fig. 1a).

**Fig. 2:**
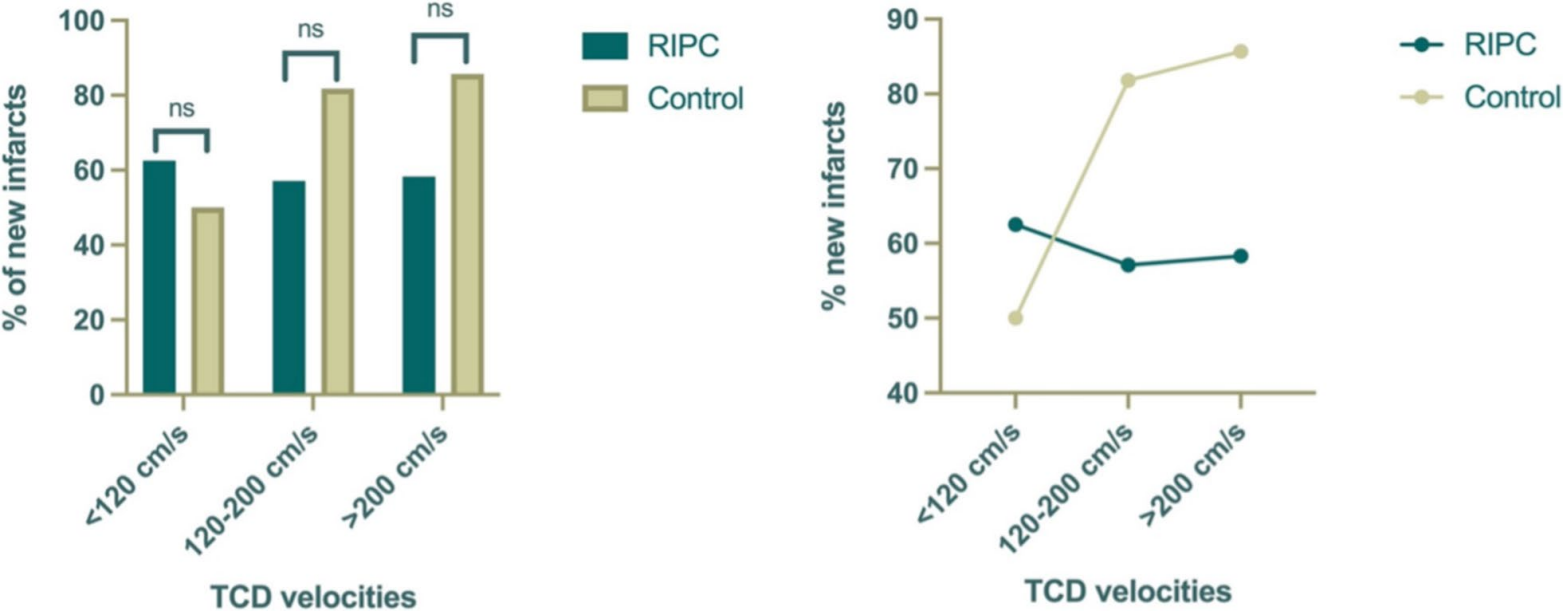
Subgroup analysis. Although the proportion of new infarcts increased in both groups as TCD velocities rose, no significant difference was found between the groups. ns, not significant; RIPC, remote ischemic preconditioning; TCD, transcranial Doppler

Altogether, seven patients underwent endovascular spasmolysis (eSL). In the intervention group, one patient required seven eSL sessions due to therapy-resistant CVS, whereas in the control group, another patient underwent three eSL sessions (Fig. 2).

In a subgroup analysis, at TCD velocities < 120 cm/s, new infarcts were observed in 62.5% of patients (*n* = 5/8) in the RIPC group and 50% of patients (*n* = 2/4) in the control group (*p* = 0.99, Fisher’s exact test). At moderate TCD elevations between 120 and 200 cm/s, new infarcts occurred in 57.1% of patients (*n* = 4/7) in the RIPC group compared with 81.8% (*n* = 9/11) in the control group (*p* = 0.32, Fisher’s exact test). For TCD velocities > 200 cm/s, new infarcts were identified in 58.3% of patients (*n* = 7/12) in the RIPC group and 85.7% (12/14) in the control group (*p* = 0.19, Fisher’s exact test).

### Secondary Outcome: Neurological Deficits

New neurological deficits occurred in 6.9% (*n* = 2) of patients in the RIPC group and in 9.6% (*n* = 3) of patients in the control group. In the RIPC group, both patients were somnolent, and one also developed aphasia; in the control group, one patient (already unconscious) showed elevated intracranial pressure, one developed aphasia, and another became confused with signs of delirium. All neurological deficits in both groups were transient and resolved by discharge or follow-up.

In terms of mortality, six patients (20.7%) in the RIPC group and four patients (12.9%) in the control group died during the hospital stay. The median time to death was 13 days (interquartile range [IQR] 10.75–14.5) in the RIPC group and 12.5 days (IQR 10.5–13.25) in the control group.

**Fig. 3:**
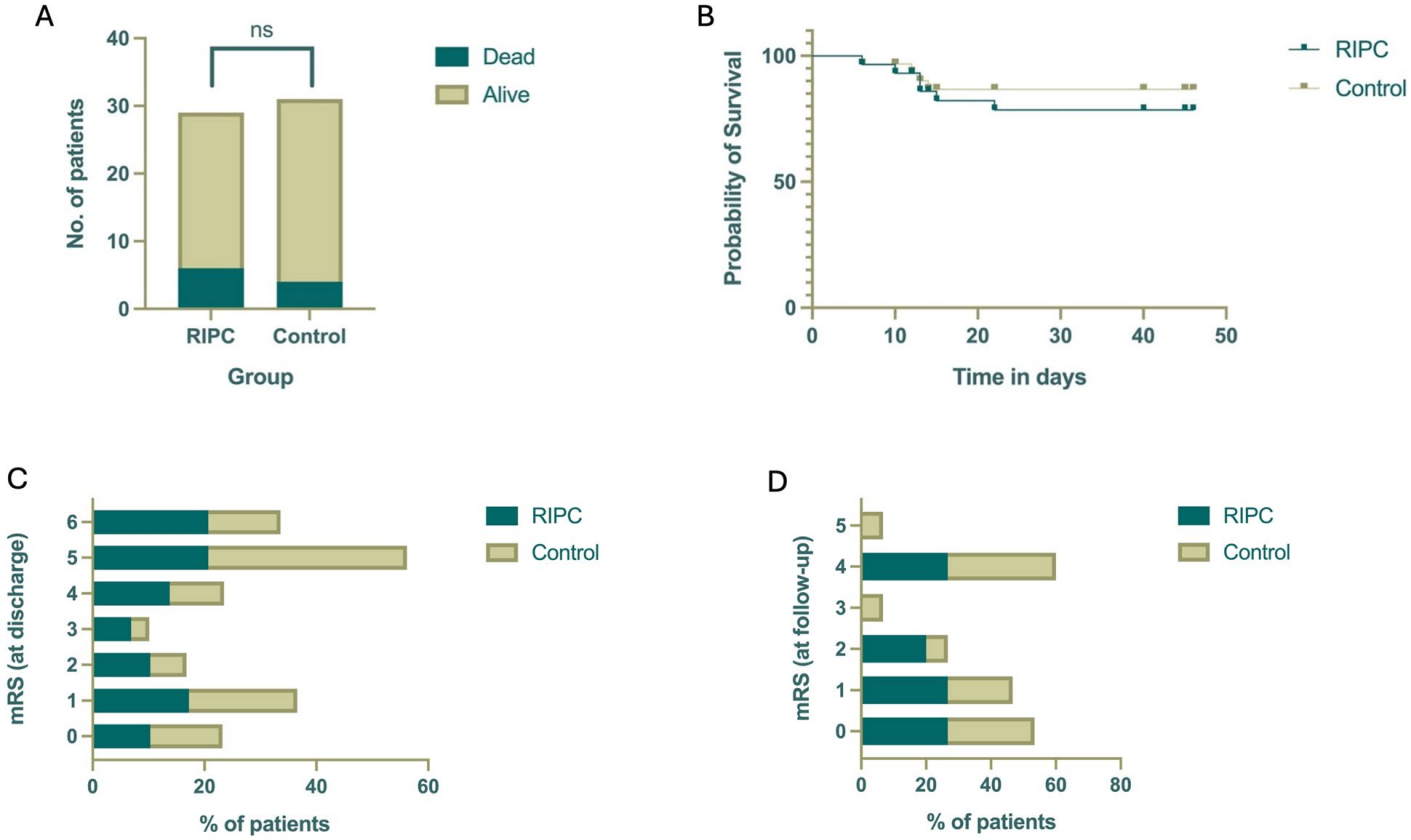
**a** Distribution of deceased and living patients across groups at discharge (*p* = 0.5) and **b** Kaplan–Meier curve for probability of survival for a 50-day hospital stay, as well as mRS at **c** discharge and **d** follow-up. mRS, modified Rankin Scale; RIPC, remote ischemic preconditioning

Follow-up data were available for 51.7% (*n* = 15) of patients in the RIPC group and 48.3% (*n* = 15) in the control group, with a median follow-up duration of 6 months in both groups (IQR 2.5 for RIPC, IQR 0.875 for control). Notably, eight patients (27.6%) in the RIPC group were not assessable for neurological deterioration during TCD monitoring due to impaired consciousness. Conclusively, there were no significant differences between the two groups with respect to incidence of new neurological symptoms (*p* = 1) or in-hospital mortality (*p* = 0.5, Fisher’s exact test, Fig. 3).

### Secondary Outcome: Functional Outcome and Disability

Regarding the disability at discharge, patients in the RIPC group showed a median mRS of 4 (IQR 1–5). Patients in the control group had a similar median mRS of 4 (IQR 1–5). At follow-up, the RIPC group presented with a median mRS of 1 (IQR 0–4). The median mRS in the control group was 2 (IQR 0–4). We observed no significant differences between the groups at discharge (*p* = 0.878, independent *t*-test) or follow-up (*p* = 0.583, independent *t*-test, Fig. 3).

However, both groups showed significantly better mRS at follow-up compared with median mRS values at discharge (RIPC *p* = 0.01, control *p* = 0.034, independent *t*-test).

## Discussion

In our study, RIPC did not seem to have a significant impact on the development and occurrence of CVS or cerebral infarctions. Although DCI is commonly defined as clinical deterioration due to cerebral ischemia, we focused on radiologically confirmed cerebral infarctions as an objective and standardized end point, as reliable clinical assessment was often limited by confounding factors such as sedation, impaired consciousness, or fluctuating neurological status. Numerous clinical studies have demonstrated the benefits of RIPC in patients in the context of cardiac surgery, particularly in reducing myocardial injury. RIPC has been shown to reduce ischemia–reperfusion damage during procedures such as coronary artery bypass grafting (CABG) and valve replacement. In such cases, the extent of myocardial injury is typically evaluated using perioperative serum levels of cardiac troponins, which act as biomarkers for myocardial infarction [9]. In a randomized controlled trial involving 57 patients undergoing elective CABG surgery, Hausenloy et al. [9] found that RIPC significantly reduced cumulative troponin T release in the first 72 h postoperatively compared with controls. Similarly, a larger, single-center, randomized trial involving 329 patients undergoing CABG surgery demonstrated that RIPC led to significantly lower perioperative concentrations of cardiac troponin I, suggesting a cardioprotective effect at the cellular level [14].

In neurosurgery, the application of RIPC has recently been explored in patients undergoing surgical resection of gliomas or brain metastases [12]. A randomized prospective trial by Sales et al. [12] involving 58 patients demonstrated that RIPC after induction of anesthesia and before surgery was associated with a reduced incidence of postoperative ischemic tissue damage as assessed by early postoperative magnetic resonance imaging (MRI) within 72 h postoperatively.

Additionally, a recent feasibility study by Koch et al. [15] involving 33 patients with aSAH investigated the safety of RIPC using limb blood pressure cuff inflations to 200 mm Hg. Although the study confirmed the safety of the intervention, it did not include outcome analysis or comparisons with a control group. In addition, the possible neuroprotective effect of RIPC on the incidence of new cerebral infarctions in patients with aSAH has not yet been studied.

RIPC could potentially provide neuroprotective effects through several mechanisms. First, it has been suggested that RIPC may improve cerebral blood flow by improving endothelial function and vasodilation, which could help reduce ischemic damage [16]. Second, RIPC may activate anti-inflammatory pathways and reduce oxidative stress, both of which play significant roles in reducing neuronal injury [17]. In addition, RIPC may trigger the release of endogenous protective molecules such as adenosine and growth factors, which could help support cellular survival and repair during cerebral ischemia [18].

In our study, 12 patients (20%) developed new cerebral infarcts on follow-up CT scans. Subgroup analysis of patients with TCD elevations exceeding 200 cm/s did not show a statistically significant difference in infarct occurrence between groups. Nevertheless, because elevated TCD velocities are generally associated with an increased risk of cerebral ischemia, further studies with larger sample sizes are needed to investigate whether RIPC might have a potential role in specific high-risk populations.

Although TCD is commonly used to detect large-vessel vasospasm in aSAH and has a high negative predictive value, its correlation with cerebral infarction and long-term outcomes are limited. Studies have shown that many patients with DCI and secondary cerebral infarcts never exhibit high TCD velocities, indicating poor sensitivity [19]. However, TCD remains a practical tool for daily monitoring and early-risk stratification in aSAH. Our findings support its continued use in this context, particularly for identifying high-risk subgroups.

The RIPC protocol used in this study consisted of three cycles of 5 min of limb ischemia and reperfusion and was based on established protocols from the previously mentioned cardiac and neurosurgical trials showing safety and feasibility. Similar dosing has also shown neuroprotective effects in animal models of cerebral ischemia with remote ischemic postconditioning after experimental stroke induction. However, the optimal ischemic dose in aSAH remains unclear. Therefore, it is possible that patients with aSAH may require a more intense or prolonged stimulus to achieve protection. Future studies should explore modified protocols to enhance efficacy while maintaining safety.

### Limitations

Our study has several limitations. First, the relatively small sample size—with only 30 patients in each group—limits the statistical power of the study. Secondly, our study did not evaluate long-term outcomes such as prolonged functional recovery or delayed complications, which could provide a more comprehensive understanding of the effectiveness of the intervention.

Another limitation is the use of CT imaging to evaluate new ischemia. MRI with diffusion-weighted imaging is a more sensitive modality for detecting ischemic regions. However, cranial CT imaging combined with CT angiography for assessing CVS is routinely performed for patients with SAH. In this intensive care cohort, it is questionable whether more complex MRI imaging with its greater strain on patient would yield more rigorous results.

Finally, subgroup analyses were performed on patients with TCD velocities of > 200 cm/s. However, no statistically significant differences in the effect of RIPC were observed. Because of the limited sample size, these exploratory analyses remain hypothesis-generating. We recommend that future studies with larger cohorts specifically investigate whether patients with elevated TCD velocities could benefit from RIPC.

In addition, because of the small sample size and optimistic assumptions about the effect size, this trial should be interpreted as an exploratory study. Although it is underpowered to detect smaller, yet still clinically relevant effects, it provides important preliminary evidence on the feasibility and potential impact of RIPC in patients with aSAH. These findings could help design larger, multicenter trials with enough statistical power.

## Conclusions

In our study, RIPC did not have a significant effect on the occurrence of vasospasms, as measured by TCD velocities, or on the development of new cerebral infarctions on CT imaging in patients with aSAH. As our primary outcome was the objective, radiologically confirmed occurrence of infarctions, these findings suggest that RIPC may have a limited impact on the prevention of ischemic brain injury in this setting. Further studies with larger cohorts are needed to better understand potential subgroup effects and underlying mechanisms.
